# Effects of Particulate Matter on Genomic DNA Methylation Content and *iNOS* Promoter Methylation

**DOI:** 10.1289/ehp.11898

**Published:** 2008-09-26

**Authors:** Letizia Tarantini, Matteo Bonzini, Pietro Apostoli, Valeria Pegoraro, Valentina Bollati, Barbara Marinelli, Laura Cantone, Giovanna Rizzo, Lifang Hou, Joel Schwartz, Pier Alberto Bertazzi, Andrea Baccarelli

**Affiliations:** 1 Laboratory of Environmental Epigenetics, Department of Preventive Medicine and Department of Environmental and Occupational Health, University of Milan and IRCCS Maggiore Hospital, Mangiagalli and Regina Elena Foundation, Milan, Italy; 2 Department of Experimental and Applied Medicine, Occupational Medicine and Industrial Hygiene, University of Brescia, Brescia, Italy; 3 Department of Preventive Medicine, Feinberg School of Medicine, Northwestern University, Chicago, Illinois, USA; 4 Exposure, Epidemiology and Risk Program, Department of Environmental Health, Harvard School of Public Health, Boston, Massachusetts, USA

**Keywords:** DNA methylation, epigenetics, etiology, interspersed repetitive sequences, nitric oxide synthase, particulate matter

## Abstract

**Background:**

Altered patterns of gene expression mediate the effects of particulate matter (PM) on human health, but mechanisms through which PM modifies gene expression are largely undetermined.

**Objectives:**

We aimed at identifying short- and long-term effects of PM exposure on DNA methylation, a major genomic mechanism of gene expression control, in workers in an electric furnace steel plant with well-characterized exposure to PM with aerodynamic diameters < 10 μm (PM_10_).

**Methods:**

We measured global genomic DNA methylation content estimated in Alu and long interspersed nuclear element-1 (LINE-1) repeated elements, and promoter DNA methylation of *iNOS* (inducible nitric oxide synthase), a gene suppressed by DNA methylation and induced by PM exposure in blood leukocytes. Quantitative DNA methylation analysis was performed through bisulfite PCR pyrosequencing on blood DNA obtained from 63 workers on the first day of a work week (baseline, after 2 days off work) and after 3 days of work (postexposure). Individual PM_10_ exposure was between 73.4 and 1,220 μg/m^3^.

**Results:**

Global methylation content estimated in Alu and LINE-1 repeated elements did not show changes in postexposure measures compared with baseline. PM_10_ exposure levels were negatively associated with methylation in both Alu [β = −0.19 %5-methylcytosine (%5mC); *p* = 0.04] and LINE-1 [β = −0.34 %5mC; *p* = 0.04], likely reflecting long-term PM_10_ effects. *iNOS* promoter DNA methylation was significantly lower in postexposure blood samples compared with baseline (difference = −0.61 %5mC; *p* = 0.02).

**Conclusions:**

We observed changes in global and gene specific methylation that should be further characterized in future investigations on the effects of PM.

Foundry work has been associated in several early investigations with adverse health outcomes, including cardiovascular and respiratory disease as well as increased risk of lung cancer [Andjelkovich et al. 1990; International Agency for research on Cancer (IARC) 1987; Kuo et al. 1999; Xu et al. 1996]. Exposures responsible for the excess in risk have not been clearly identified (IARC 1987). In modern foundry facilities, exposures to chemicals are remarkably lower than in the past (Bergamaschi et al. 2005), but particulate matter (PM) levels are still well above the concentrations found in ambient outdoor air. Ambient PM has also been associated with increased hospitalization and mortality due to cardiorespiratory disease and lung cancer (Brook et al. 2004; Peters 2005; Samet et al. 2000; Vineis and Husgafvel-Pursiainen 2005). Epidemiologic (Brook et al. 2004; Peters 2005; Schulz et al. 2005) and *in vivo* studies (Chang et al. 2005; Chen and Hwang 2005; Corey et al. 2006) suggest that the transition metal components of PM may be responsible for such effects.

The mechanisms linking PM inhalation to adverse health outcomes have not been completely clarified. Inhaled particulate pollutants have been shown to produce systemic changes in gene expression, which can be detected in peripheral blood of exposed individuals (Wang et al. 2005). Gene expression of human genes is controlled by DNA methylation, which, in mammals, involves the postreplication addition of methyl groups to the 5′ position of cytosine ring within the context of CpG dinucleotides to form 5-methylcytosine (5mC). Initial observations of *in vitro* and animal models have shown that air particles, or air particle components such as toxic metals, can induce changes in DNA methylation (Belinsky et al. 2002; Takiguchi et al. 2003). Whether DNA methylation changes occur in human subjects exposed to PM has never been determined.

Reduced genomic methylation content in blood DNA has been observed in subjects with cardiovascular disease, as well as in cancer subjects (Robertson 2005). Genomic DNA hypomethylation is likely to result from demethylation in transposable repetitive elements, which plays a crucial role in gene regulation and genomic stability. More than 90% of all genomic 5-methylcytosines lies within CpG islands located in transposable repetitive elements, including Alu and long interspersed nuclear element-1 (LINE-1) sequences, which are those most common and well characterized. Measurements of Alu and LINE-1 methylation have been used to estimate global genomic DNA methylation content (Yang et al. 2004). *In vitro* studies have shown that reactive oxygen species (ROS), which are considered one of the main cellular stressors generated by PM exposure (Borm et al. 2007), may produce genomic hypomethylation (Valinluck et al. 2004). Conditions associated with reduced global DNA methylation content, such as specific dietary and genetic variations (Friso and Choi 2002; Friso et al. 2002), have been shown to interact with ambient PM exposure to produce health-related outcomes (Baccarelli et al. 2008).

Elevated expression of the inducible nitric oxide synthase gene *(iNOS*, also known as *NOS2,* Genbank accession number AF017634) has been observed in animal experiments of exposure to PM or PM components in the lung and across other different tissues (Folkmann et al. 2007; Thomson et al. 2007; Ulrich et al. 2002), including blood leukocytes (Blackford et al. 1994). Specific studies on *iNOS* have shown that lower DNA methylation in the gene promoter is associated with increased expression (Chan et al. 2005). *iNOS* expression and activity are increased in the presence of ROS (Zhen et al. 2008) and other factors, such as cigarette smoke (Anazawa et al. 2004; Chyu et al. 1999; Wright et al. 1999), associated with cardiorespiratory outcomes.

In the present work, we investigated short- and long-term effects of particle exposure on DNA methylation in peripheral blood DNA from workers with well-characterized exposure to a wide range of PM levels in an electric steel furnace plant. We measured global genomic DNA methylation content, estimated in Alu and LINE-1 repetitive elements*,* and promoter methylation of *iNOS.*

## Material and Methods

### Study subjects

We recruited 63 healthy, male workers (mean age 44 years; range between 27 and 55 years) free of cardiovascular and pulmonary disease in a steel production plant in Brescia, Northern Italy. All participants had been working in the present job position for at least 1 year. Thirty-seven subjects had a rotating weekly schedule based on four consecutive working days of 8 hr each, followed by 2 days of rest. The remaining 26 subjects worked Monday through Friday, also in 8-hr shifts. Twenty-five subjects (40%) were current smokers, who reported a mean (±SD) number of 13.0 ± 7.2 cigarettes smoked every day. The average body mass index of the study participants was 26.5 ± 2.7 kg/m^2^.

A self-administered questionnaire was used to collect detailed information on lifestyle, drug use, recent medical conditions, and residential history. Records from the factory administrative and clinical files were used to abstract information on occupational and past medical history.

In order to discriminate short- and long-term effects of PM, we obtained blood samples for DNA methylation analysis at two different times. Sample 1 was collected in the morning of the first day of work (after 2 days off work) before the beginning of any work activity. Sample 2 was collected at the same hour on the fourth day of work, after 3 consecutive days of work. Individual written informed consent and approval from the local Institutional Review Board were obtained before the study.

### Exposure assessment

Measures of PM with aerodynamic diameters < 10 μm (PM_10_) obtained in each of the 11 work areas of the steel production plant were used to estimate individual exposures. PM_10_ was measured during the days between sample 1 and sample 2 collection using a GRIMM 1100 light-scattering dust analyzer (Grimm Technologies, Inc. Douglasville, GA, USA).

During the 3 working days between blood samples 1 and 2, each of the study subjects recorded in a personal log the time he spent in each of the work areas. Individual exposure was calculated as the average of PM_10_ weighted by the time spent in each area. PM_10_ levels in each of the work areas have shown very little variability over time, as measures repeated over 1 year showed very high correlation between PM_10_ concentrations (*r*^2^ > 0.90). Because all the study subjects reported in the questionnaire to have performed their standard work routine during the 3 days of the study, the time-weighted PM_10_ represented, in addition to the exposure during the week of the study, a measure of the usual exposure of the study subjects. Therefore, we considered our study subjects to be usually exposed to the levels of PM_10_ measured during the week of the examination. In the statistical analysis, as specified below, we evaluated the association of PM_10_ levels with *a*) DNA methylation measured at the end of the work week (sample 1), which, together with short-term changes between sample 1 and sample 2, were taken as measures of short term effects; *b*) DNA methylation measured at the beginning (sample 1) and at the end of the work week (sample 2), which were analyzed jointly in repeated measure analysis and taken as a measure of long-term effects.

### DNA methylation analysis

We used EDTA tubes to collect 7 mL whole blood that was promptly centrifuged on site at 2,500 rpm for 15 min. The buffy coat (400 μL) was transferred in a cryovial, immediately frozen in vapor phase of liquid nitrogen, and shipped in nitrogen dry shippers to the laboratory. DNA was extracted using the Wizard Genomic DNA purification kit (Promega, Madison, WI, USA) following the manufacturer’s instructions.

We performed DNA methylation analyses on bisulfite-treated DNA using highly quantitative analysis based on PCR pyrosequencing; 1 μg DNA (concentration 50 ng/μL) was treated using the EZ DNA Methylation-Gold Kit (Zymo Research, Orange, CA, USA) according to the manufacturer’s protocol. Final elution was performed with 30 μL M-Elution Buffer.

To estimate global DNA methylation content, we performed DNA methylation analyses of Alu and LINE-1 repeated sequences, which allow for the amplification of a representative pool of repetitive elements, as previously described (Bollati et al. 2007). Measures of Alu and LINE-1 methylation have been shown to be highly correlated with 5-methylcytosine content measured through high performance liquid chromatography and are commonly used as a surrogate of global methylation (Weisenberger et al. 2005; Yang et al. 2004).

We developed the assay for *iNOS* methylation by locating the *iNOS* promoter using the Genomatix Software (Genomatix Software Inc, Ann Arbor, MI, USA) on chromosome 17 (start = 23149861, end = 23150461), and amplified the sequence between 23149872 and 23149990. A 50-μL PCR was carried out in 25 μL GoTaq Green Master mix (Promega), 10 pmol forward primer, 10 pmol reverse primer, 50 ng bisulfite-treated genomic DNA, and water. PCR cycling conditions were 95°C for 30 sec, 50°C for 30 sec, and 72°C for 30 sec for 40 cycles. PCR products were purified and sequenced by pyrosequencing as previously described (Bollati et al. 2007) using 0.3 μM sequencing primer.

Primers for Alu, LINE-1, and *iNOS* assay are shown in Table 1. For all assays we used built-in controls to verify bisulfite conversion efficiency. Compared with other common methods of DNA methylation analysis, pyrosequencing-based assays have the advantage of producing individual measures of methylation at more than one CpG dinucleotide, thus reflecting more accurately DNA methylation in the region. In the Alu or LINE-1 assays, we measured the percentage of 5mC (%5mC) at each of three CpG dinucleotide positions that are repeated over the human genome with the sequence of interest.

In the *iNOS* promoter assay, we measured %5mC at each of two individual CpG dinucleotides within a CpG island located in the gene promoter.

The within-sample coefficients of variation were 0.7% for LINE-1, 1.6% for Alu, and 0.7% for *iNOS*. The between-sample coefficients of variation in this study population were 1.7% for LINE-1, 3.3% for Alu, and 5.3% for *iNOS*. Every sample was tested two times for each assay to confirm reproducibility. The resulting data were analyzed using mixed models, as described in the statistical analysis section below.

### Statistical analysis

In each blood sample, the pyrosequencing-based analysis of DNA methylation produced six values each for Alu or LINE-1 (methylation at three CpG dinucleotide positions replicated in two measurements) and four values for *iNOS* (methylation at two individual CpG dinucleotide positions replicated in two measurements). Each subject was tested twice [at the beginning of the work week (sample 1) and after 3 days of work (sample 2)]. To account for the data structure, we used mixed effects models, as described below.

### Analysis of short-term effects of PM exposure on DNA methylation

We first evaluated differences between sample 1 and sample 2 in two-way crossed random effects models:


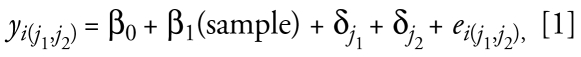


where β_0_ is the overall intercept; β_1_ is the regression coefficient for the difference between Samples 1 and 2; *j*_1_ represents the subject; *j*_2_ represents the CpG dinucleotides position; δ_*j*_1__ is the random effect for subject *j*1; δ_*j*_2__ is the random effect for CpG dinucleotides position; and e_i(*j*_1_,*j*_2_)_ is the residual error term. Likelihood ratio tests were used to test for the significance of β_1_.

We then evaluated whether DNA methylation measured after 3 days of work (Sample 2) was associated with the PM_10_ exposure level estimated during the previous 3 days, using two-way crossed random effects models:


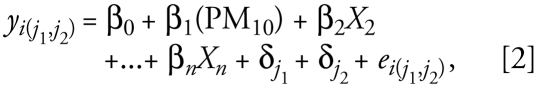


where β_0_ is the overall intercept; β_1_ is the regression coefficient for PM_10_ exposure; β_2_… β*_n_* are the regression coefficients for the covariates included in multivariate models; *j*_1_ represents the subject; *j*_2_ represents the CpG dinucleotides position; δ_*j*_1__ is the random effect for subject *j*_1_; δ_*j*_2__ is the random effect for CpG dinucleotides position, and e_i(*j*_1_,*j*_2_)_ is the residual error term. Covariates for multivariate models included the following potential confounders that were chosen *a priori* and included in the analysis: age, body mass index, smoking, and number of cigarettes/day. These variables were not significantly associated in univariate analysis with methylation of Alu (β = 0.00, SE = 0.01, *p* = 0.99 for age; β = 0.01, SE = 0.03, *p* = 0.83 for BMI; β = 0.11, SE = 0.15, *p* = 0.46 for smoking; β = −0.01, SE = 0.01, *p* = 0.73 for cigarettes/day); LINE-1 (β = 0.00, SE = 0.02, *p* = 0.94 for age; β = −0.06, SE = 0.05, *p* = 0.19 for BMI; β = −0.15, SE = 0.27, *p* = 0.57 for smoking; β = −0.02, SE = 0.03, *p* = 0.41 for cigarettes/day) or *iNOS* (β = −0.03, SE = 0.06, *p* = 0.57 for age; β = −0.19, SE = 0.16, *p* = 0.23 for BMI; β = 0.47, SE = 0.89, *p* = 0.59 for smoking; β = −0.02, SE = 0.09, *p* = 0.82 for cigarettes/day).

### Analysis of long-term effects of PM exposure on DNA methylation

As noted in the exposure assessment section, PM_10_ exposure levels estimated during the study also represented a measure of the usual exposure of the study subjects. To estimate long-term effects of PM_10_ on DNA methylation, we evaluated the level of individual exposure to PM_10_ in relation to all the measures of DNA methylation performed in the study, regardless of whether they were measured on samples taken on the first day of work (sample 1), or after 3 consecutive days of exposure to PM_10_ (sample 2), thus assuming that PM_10_ effects operating over an extended time frame produced similar modifications at the two time points.

For DNA methylation measures that did not show changes in the analysis of short-term effects, we fit two-way error-components models, as described in the formula [2] above. If a significant difference between samples 1 and 2 was found in the analysis of short-term effects, we fit a three-way error-components model*,* as described in the following notation:


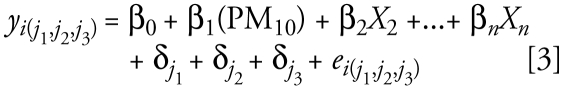


where β_0_ is the overall intercept; β_1_ represents the mean PM_10_ effect; β_2_… β*_n_* are the regression coefficients for the covariates included in multivariate models; *j*_1_ represents the subject; *j*_2_ represents the CpG dinucleotide; and *j*_3_ represents the blood sample (sample 1 or 2); δ_*j*_1__ is the random effect for subject *j*1; δ_*j*_2__ is the random effect for the CpG dinucleotide position *j*_2_; and δ_*j*_3__ is the random effect for blood sample *j*_3;_ *e_i_*_(*j*_1_,*j*_2_*,j*_3_)_ is the residual error term.

Covariates for multivariate models included the same variables as in the analysis of short-term effects (age, body mass index, smoking, and number of cigarettes/day).

## Results

### Distribution of DNA methylation data

DNA methylation showed changes among different blood DNA samples that were relatively small compared with the mean methylation. DNA methylation in Alu repeated elements ranged between 24.3 and 28.9 %5mC, with a mean of 25.8 %5mC (SD = 0.83). DNA methylation in LINE-1 repeated elements ranged between 75.9 and 86.1 %5mC, with a mean of 78.8 %5mC (SD = 1.22). DNA methylation in *iNOS* ranged between 56.2 and 75.6 %5mC, with a mean of 67.8 %5mC (SD = 3.52).

### Short-term effects of PM_10_ exposure on DNA methylation

Individual PM_10_ average levels estimated for each subject during the 3 work days between the first and the second DNA methylation measurement (sample 1 and sample 2) ranged between 73.4 and 1220.2 (μg/m^3^) (average 233.4 μg/m^3^). As shown in Table 2, DNA methylation of Alu and LINE-1 repeated elements did not show any change after 3 days of work (sample 2) compared with the baseline measurements taken at the beginning of the first day of work (sample 1) (mean difference = 0.00 %5mC, SE = 0.08, *p* = 0.99 for Alu; mean difference = 0.02 %5mC, SE = 0.11, *p* = 0.89 for LINE-1). DNA methylation in the *iNOS* promoter was significantly decreased after 3 days of work compared with the baseline measurement (mean difference = −0.61 %5mC; SE = 0.26, *p* = 0.02) (Table 2). The average level of individual exposure to PM_10_ during the 3 days of work showed negative correlations with DNA methylation of Alu*,* LINE-1, and *iNOS* measured in sample 2 (Table 3), with associations that were not statistically significant in unadjusted analysis, as well as in models adjusted for age, body mass index, smoking, and number of cigarettes/day.

### Long-term effects of PM_10_ exposure on DNA methylation

To identify possible long-term effects of PM_10_ exposure, we evaluated the level of individual exposure to PM_10_, taken as a measure of usual exposure to particles, in relation to all the measures of DNA methylation performed in the study, regardless of whether they were measured on samples taken on the first day of work (i.e., after 2 days off, sample 1), or after 3 consecutive days of work (sample 2) (Table 4). In the models, the two samples collected at different times are exchangeable, thus assuming that PM_10_ effects operating over an extended time frame produced similar modifications at the two time points.

**Figure 1:**
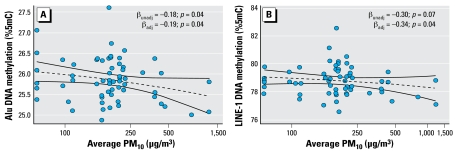
Long-term effects of PM_10_ on blood DNA methylation in Alu (*A*) and LINE-1 (*B*) repeated elements. PM_10_ concentrations, taken as a measure of usual exposure to particles, was examined in relation to all the measures of DNA methylation performed in the study, regardless of whether they were measured on samples taken on the first day of work (i.e., after 2 days off) or after 3 consecutive days of exposure to PM_10_ in the plant. Abbreviations: adj, adjusted; nonadj, nonadjusted. Data points represent the average of DNA.

In unadjusted models, the average PM_10_ levels were significantly associated with decreased Alu methylation (β = −0.18, SE = 0.09; *p* = 0.04). A negative, nonsignificant association was also observed for LINE-1 methylation (β = −0.30, SE = 0.17, *p* = 0.07). In multivariable regression analysis adjusting for age, body mass index, smoking, and number of cigarettes, the average PM_10_ levels were significantly and negatively associated with both Alu (β = −0.19, SE = 0.09, *p* = 0.04) and LINE-1 (β = −0.34, SE = 0.17, *p* = 0.04) methylation. Scatter plots representing the association of average PM_10_ level with Alu and LINE-1 methylation are shown in Figure 1.

*iNOS* methylation showed no association with average PM_10_ level in both non-adjusted (β *=* −0.48, SE = 0.58, *p* = 0.41) and multivariable analyses (β *=* −0.55, SE = 0.58, *p*= 0.34) (Table 4).

In addition, we also evaluated the association of PM_10_ level with DNA methylation measured on blood DNA collected on the first day of the work week (Sample 2), as long-term effects of the exposure would likely be reflected also on samples collected after 2 days off work. The associations found between average PM_10_ levels and DNA methylation measured at the beginning of the first week were in the same directions as those in the primary analysis reported in Table 4 both in the unadjusted (β *=* −0.18 SE = 0.12, *p* = 0.14 for Alu; β *=* −0.34, SE = 0.16, *p* = 0.04 for LINE-1; β *=* −0.66, SE = 0.60 *p* = 0.24 for *iNOS*) and multivariable analysis (β *=* −0.19, SE = 0.12, *p* = 0.11 for Alu; β *=* −0.39, SE = 0.15, *p* = 0.01 for LINE-1; β *=* −0.67, SE = 0.59, *p* = 0.25 for *iNOS*).

## Discussion

In the present study of workers in an electric furnace steel plant with well-characterized measures of exposure to a wide range of PM_10_ levels, global DNA methylation estimated in Alu and LINE-1 repeated elements were negatively associated with individual PM_10_ exposure, without changes related to short-term exposure during the week of the study. We observed short-term changes in *iNOS* promoter methylation, which decreased after 3 consecutive days of work in the plant.

Decreases in global DNA methylation content have been associated with widespread alterations in gene expression and chromatin packaging control, as well as with higher genomic instability (Dean et al. 2005). The decrease we observed in our study in association with PM_10_ exposure may represent an initial step reproducing decreases in global DNA methylation content that are eventually observed in cardiovascular disease and cancer (Baccarelli et al. 2007; Robertson 2005). In our study, the association between PM_10_ level and decreased methylation in Alu and LINE-1 was significant only when the two measurements of methylation taken before and after 3 consecutive work days, which showed no differences in Alu and LINE-1 methylation, were both included in repeated-measure models. In our analyses, the use in the same models of both methylation measures taken at the beginning and at the end of the work week was meant to evaluate long-term effects of PM_10_, which would have similar effects on measures of DNA methylation taken at the two different time points, and also provided our statistical analysis with added power to detect the PM_10_ effects. These results suggest that PM_10_ operated on genomic DNA methylation content over an extended time frame, possibly causing a persistent suppression of methylation levels that were not reset to baseline over the 2 days off between consecutive work weeks.

Air particle exposure has been shown to cause increased *iNOS* expression in animal models (Folkmann et al. 2007; Ulrich et al. 2002). In our study, *iNOS* methylation, which has been previously shown to keep *iNOS* expression suppressed (Chan et al. 2005), was significantly decreased after 3 days of work, compared with measures taken before the first day of work of the same week. However, we did not find any association with levels of PM_10_ exposure; thus, whether *iNOS* promoter methylation is modified by short-term exposure to PM remains uncertain. *In vitro* studies have shown that methylation of individual genes undergoes rapid changes in response to environmental factors (Bruniquel and Schwartz 2003; Takiguchi et al. 2003), and *iNOS* expression has been found to respond rapidly to different stimuli, including immunostimulatory cytokines, bacterial products, or infection (Alderton et al. 2001). Increased *iNOS* expression has been found in disease conditions that have also been associated with PM exposure, such as cardiovascular disease and lung cancer (Comini et al. 1999; Liu et al. 1998). Whether *iNOS* expression is increased after PM exposure due to *iNOS* promoter demethylation should be clarified in subjects exposed acutely to particles after an extended washout period, as well as in larger populations of exposed subjects. Future investigations should also aim at clarifying whether changes in *iNOS* promoter methylation modify its expression, as *iNOS* expression was not measured in this study.

Although several other genes might have been included in our study, we selected *iNOS* for DNA methylation analysis because the increase of expression after exposure to PM or PM components has been well substantiated in previous studies conducted on several tissues (Anazawa et al. 2004; Blackford et al. 1997; Castranova 2004; Folkmann et al. 2007; Porter et al. 2006) including blood leukocytes (Blackford et al. 1994), which were the source of DNA for our study. Further research is warranted to evaluate PM-related changes in DNA methylation of *iNOS-*related genes, as well as in other independent pathways.

Our study was based on quantitative analysis of DNA methylation using pyrosequencing, which is highly reproducible and accurate at measuring small changes in DNA methylation (Bollati et al. 2007; Yang et al. 2004). DNA methylation analysis measured multiple individual CpG dinucleotide positions for each marker and was repeated twice on each sample to minimize the assay variability. We used multilevel mixed models to fully represent the structure of the data and take advantage of the multiple measurements while also adjusting for potential confounders. Our data comprised each of the methylation markers investigated (Alu, LINE-1, and *iNOS*) of a matrix of six or four measures (3 × 2 or 2 × 2), including data from three (for Alu and LINE-1) or two (for *iNOS*) CpG dinucleotides and from two replicates. Commonly used statistical methods for the analysis of such data would include linear regression and analysis of variance, using as the outcome the mean computed from the multiple CpG dinucleotides and replicates from each sample. However, because the data in the methylation matrix obtained on an individual sample were not independent, the use of standard methods would not adequately represent the correlation existing within the matrix. We therefore elected to use multilevel mixed models that allowed us to fully utilize the information from all the measurements in our data and maximize statistical power by distinguishing between the different sources of variance in the data.

We investigated a population with well-characterized PM_10_ exposure that allowed for contrasting subjects over a wide range of different exposure levels. Our study was based on subjects working in several work areas of the same factory but did not include a different population of subjects without a specific condition of exposure to PM. However, the lowest level of PM_10_ observed in our study (74 μg/m^3^) was relatively low, particularly if compared with the highest level found in our population (1,220 μg/m^3^), and only marginally higher than ambient PM_10_ levels measured in the geographic area in which the plant is located [average annual ambient PM_10_ levels between 41 and 57 μg/m^3^ were recorded in the year of the study by different ambient monitoring stations in the Brescia area (Anselmi and Patelli 2006)]. In addition, limiting our investigation to individuals who have all been working in the same work facility avoided potential concerns related to the selection of external referents who might have differed from the exposed population in terms of socioeconomic factors and other characteristics determining hiring into the plant (Pearce et al. 2007).

In addition to PM, workers in foundries may have additional exposures, including heat, polycyclic aromatic hydrocarbons (Mirer 1998; Sorahan et al. 1994), carbon monoxide (Lewis et al. 1992; Park 2001), and non-ionizing radiations (Gomes et al. 2002). Although study subjects in our study were in a modern facility with state-of-the-art systems for exposure reduction, we cannot exclude that exposures other than PM might have contributed to the observed effects.

Our results showed alterations in blood DNA methylation in a population of foundry workers, including changes in global methylation estimated in Alu and LINE-1 repetitive elements and gene-specific methylation of the *iNOS* promoter. Further studies are required to determine the role of such alterations in mediating the effects of particles on human health.

## Figures and Tables

**Table 1:** Primers for DNA methylation analysis.

Sequence ID	Forward primer (5′ to 3′)	Reverse primer (5′ to 3′)	Sequencing primer (5′ to 3′)	Sequence analyzed^a^
Global methylation analysis				
Alu	Biotin-TTTTTATTAAAAATATAAAAATT	CCCAAACTAAAATACAATAA	AATAACTAAAATTACAAAC	G/AC/TG/AC/TG/ACCACCA
LINE-1	TTTTGAGTTAGGTGTGGGATATA	Biotin-AAAATCAAAAAATTCCCTTTC	AGTTAGGTGTGGGATATAGT	TTC/TGTGGTGC/TGTC/TG
Gene-specific methylation analysis				
*iNOS*	AATGAGAGTTGTTGTTGGGAAGTGTTT	Biotin-CCACCAAACCCAACCAAACT	TAAAGGTATTTTTGTTTTAA	C/TGATTTTC/TGGGTTTTTTTTTATTTTG

a Nucleotides at which DNA methylation was measured are underlined

**Table 2:** Change in methylation of Alu, LINE-1, and *iNOS,* after 3 days of work (sample 2) compared with measures on the first day of work (sample 1).

		Mean DNA methylation		Difference in DNA methylation (Sample 2 – Sample 1)		
	No.of subjects	Sample 1	Sample 2	Mean	SE	*p*-Value
Global methylation analysis						
Alu (%5mC)	61	25.8 (0.7)	25.8 (0.6)	0.00	0.08	0.99
LINE-1 (%5mC)	61	78.8 (1.0)	78.8 (1.5)	0.02	0.11	0.89
Gene-specific methylation analysis						
*iNOS* (%5mC)	60	68.8 (3.5)	68.2 (3.7)	−0.61	0.26	0.02

**Table 3:** Association of PM_10_ average exposure with methylation of Alu, LINE-1, and *iNOS* measured after 3 consecutive work days of exposure.

	Unadjusted regression			Adjusted regression^a^		
	β^b^	SE	*p*-Value	β^b^	SE	*p*-Value
Global methylation analysis						
Alu	−0.18	0.10	0.08	−0.18	0.10	0.071
LINE-1	−0.25	0.25	0.31	−0.28	0.25	0.26
Gene-specific methylation analysis						
*iNOS*	−0.27	0.63	0.66	−0.39	0.62	0.53

a Multivariable regression models adjusted for age, body mass index, smoking, number of cigarettes/day.

b β for an increment equal to the difference between the 90th and 10th percentile of PM10.

**Table 4:** Association of PM_10_ average level with all measures of DNA methylation in blood samples taken from exposed workers.^a^

	Unadjusted regression			Adjusted regression^b^		
	β^c^	SE	*p*-Value	β^c^	SE	*p*-Value
Global methylation analysis						
Alu	−0.18	0.09	0.04	−0.19	0.09	0.04
LINE-1	−0.30	0.17	0.07	−0.34	0.17	0.04
Gene-specific methylation analysis						
*iNOS*	−0.48	0.58	0.41	−0.55	0.58	0.34

a To estimate long-term effects of PM10, the level of individual exposure to PM10, taken as a measure of usual exposure to particles, was examined in relation to all the measures of DNA methylation performed in the study, regardless of whether they were measured on samples taken on the first day of work (i.e., after 2 days off) or after 3 consecutive days of exposure to PM10 in the plant. In the models, the two samples collected at different times are exchangeable, thus assuming that PM10 effects operating over an extended time frame produced similar modifications at the two time points.

b Multi-variable mixed models adjusted for age, body mass index, smoking, number of cigarettes/day.

c β for an increment equal to the difference between the 90th and 10th percentile of PM10.
